# Refining drug administration in a murine model of acute infection with *Trypanosoma cruzi*

**DOI:** 10.1186/s42826-020-00071-z

**Published:** 2020-10-20

**Authors:** Julián Ernesto Nicolás Gulin, Margarita Bisio, Facundo García-Bournissen

**Affiliations:** 1 Instituto Multidisciplinario de Investigaciones en Patologías Pediátricas (IMIPP), Hospital de Niños “Dr. Ricardo Gutiérrez”, CONICET- GCBA, Gallo 1330, 1425 Buenos Aires, Argentina; 2 Servicio de Parasitología y Enfermedad de Chagas, Hospital de Niños “Dr. Ricardo Gutiérrez”. Ministerio de Salud. GCBA, Buenos Aires, Argentina

**Keywords:** Chagas disease animal models, Chronic treatment - Oral administration, Preclinical drug research, Refinement

## Abstract

**Background:**

In animal research, “refinement” refers to modifications of husbandry or experimental procedures to enhance animal well-being and minimize or eliminate pain and distress. Evaluation of drug efficacy in mice models, such as those used to study *Trypanosoma cruzi* infection, require prolonged drug administration by the oral route (e.g. for 20 consecutive days). However, the orogastric gavage method can lead to significant discomfort, upper digestive or respiratory tract lesions, aspiration pneumonia and even accidental death. The aim of this work was to evaluate the effect of two administration methods (conventional oral gavage vs. a refined method using a disposable tip and automatic pipette) on the efficacy of benznidazole in a murine model of *T. cruzi* infection.

**Results:**

Both administration methods led to a rapid and persistent reduction in parasitaemia. Absence of *T. cruzi* DNA (evaluated by real-time PCR) in blood, cardiac and skeletal muscle confirmed that treatment efficacy was not influenced by the administration method used.

**Conclusions:**

The proposed refined method for long-term oral drug administration may be a suitable strategy for assessing drug efficacy in mice models of Chagas disease and can be applied to similar murine infection models to reduce animal discomfort.

## Background

Drug administration by the oral route is a common method to achieve systemic exposure. Substances can be delivered to the gastrointestinal tract by including them in water or food, by oropharyngeal administration of capsules, pills or fluids, or through gavage [1] requiring physical restraint by trained staff to minimize animal harm and stress.

When a research protocol requires chronic oral drug administration, inclusion in food or water may be possible, but may increase variability and uncertainty in the ingested dose, reducing dosing accuracy. In addition, many compounds are not water-soluble, and environmental conditions such as temperature and humidity can affect stability [2].

Oral gavage delivers a known drug amount in a single administration step. Though effective, it requires substantial technical skills, is very labor-intensive and is not suitable for long-term and/or frequent treatments. Disadvantages of gavage dosing include risks of esophageal or stomach damage and inadvertent administration into the airway using both house-made and commercial devices systems [3].

In animal research, “refinement” refers to “modifications of husbandry or experimental procedures to enhance animal well-being and minimize or eliminate pain and distress” [4]. While many efforts have been done to refine post-surgical analgesia administration [5, 6] to minimize stress interference in behavior studies [7] or in neuroleptic therapy models [8] there is still a lack of information on the bias that less invasive oral administration methods may introduce in parasitology models.

Treatment options for Chagas disease rely on benznidazole (BZ) and nifurtimox (NFX), developed over four decades ago and still the only available chemotherapy, with potential severe adverse effects. Thus, there is an urgent need for new and better chemotherapy for this endemic parasitic disease [9].

In this context, a consensus for pre-clinical drug discovery for Chagas disease has emerged, which proposes a 20 consecutive day treatment protocol using the oral route to evaluate the efficacy of new anti-*Trypanosoma cruzi* compounds in the murine model of acute infection [10].

To alleviate potential drawbacks of the gavage method, we developed a refined method of oral administration using a disposable tip and automatic pipette. The aim of this work is to compare both oral administration methods (i.e. oral gavage vs. the refined method using a disposable tip with an automatic pipette) on the efficacy of BZ in a murine model of *T. cruzi* infection during a twenty-consecutive day protocol.

## Results

### Animal welfare

Benznidazole treatment was well tolerated by mice throughout the study whatever the administration method. Weight and body temperature were registered regularly and there were no statistical differences between both administration methods (data not shown). Mice receiving treatment through traditional oral gavage displayed aversion to the procedure and the discomfort lasted for the 10–15 min after dosage (noted as immobility, gagging and respiratory distress). Conversely, the refined method was well accepted and all mice received the full volume of the drug. Mice treated with the refined method seemed normal and resumed their usual behavior immediately after drug administration. There was no damage observed on the hard palate during or immediately after drug administration and there no substance loss during the treatment. All mice survived the treatment period in both administration methods.

### Treatment and assessment of cure

**Fig. 1:**
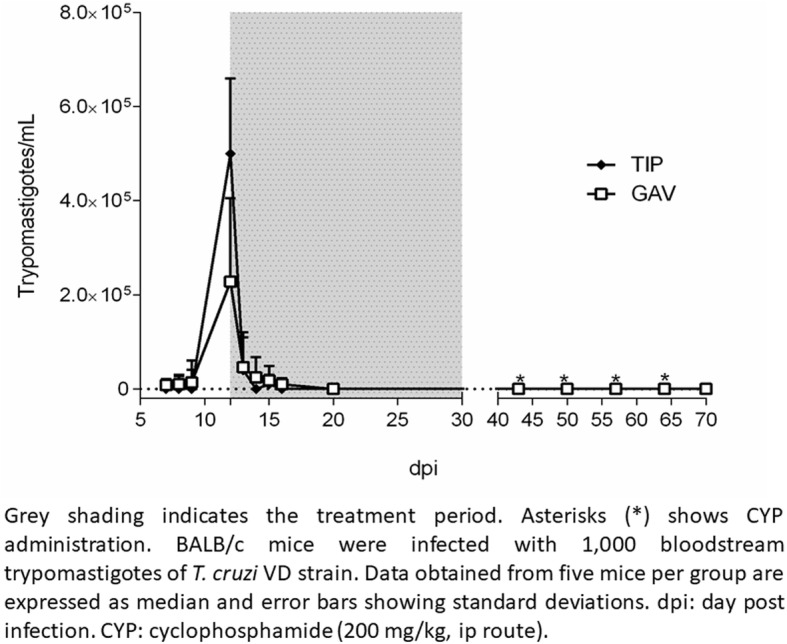
Parasitaemia curve in infected mice treated with benznidazole using gavage (GAV) or tip method (TIP)

Parasitaemia was evaluated daily starting on the 7th day post-infection (dpi) by direct microscopy. All infected animals displayed patent parasitaemia at 12 dpi, and BZ treatment started immediately (Fig. 1). Since Shapiro-Wilk tests analysis showed that parasitaemia values did not approximate to normality (*p* = 0.43 for TIP *p* = 0.53 for GAV), nonparametric tests were used for analysis.

Acute infection was established similarly in both groups. Although at treatment onset TIP group had significantly higher mean parasitaemia values than GAV (4.84 × 10^5^ vs. 2.28 × 10^5^; *p* = 0.03; Mann-Whitney) parasitaemia course was similar between experimental groups and as expected with the acute phase of infection by the *T. cruzi* VD strain (Fig. 1).

A median of 4 BZ doses (range 1 to 5 doses) were needed to reach parasitaemia clearance in the TIP administration group, while and in the GAV group, a median of 5 BZ doses were required (range 1 to 5 doses). The number of doses to achieve parasitaemia clearance were not statistically different (*p* = 0.59; Mann Whitney test). At the end of treatment, (i.e., 31 dpi), all animals had negative parasitaemia by direct blood observation and no mortality was observed in any group.

**Fig. 2:**
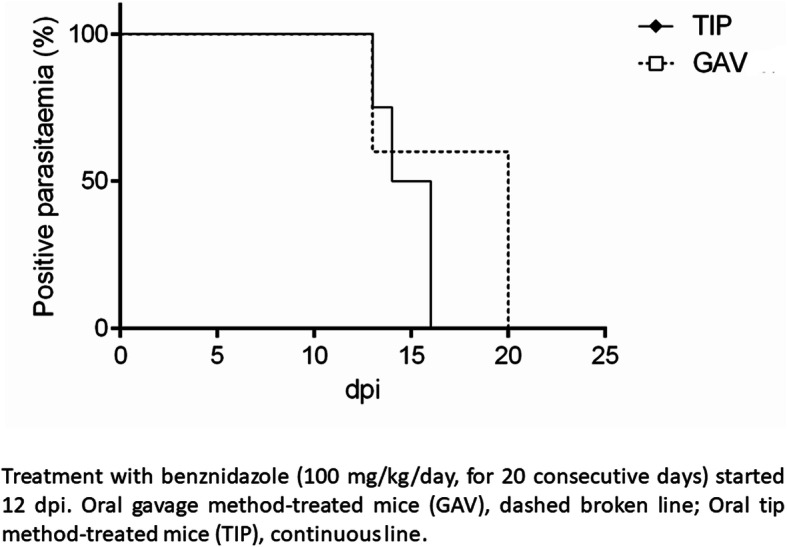
Cumulative median time to parasitaemia clearance in mice infected with* Trypanosoma cruzi*

Similarly, no differences were detected in median patent parasitaemia period between both administration methods: 4 days for TIP group (range 1 to 9 days) and 8 days for GAV group (range 1 to 8 days) (*p* = 0.88; Mantel-Cox log rank sum) (Fig. 2).

Immunosuppression cycles started at 43 dpi and were repeated at 50, 57 and 64 dpi. As no mice developed rebound parasitaemia by direct blood examination, parasitological cure was explored by qPCR in blood, heart, and skeletal muscle (Table 1). All heart samples but one (from GAV group) were negative while skeletal muscle samples were negative or below the limit of quantitation by qPCR therefore the animals were considered cured.

**Table 1:** *Trypanosoma cruzi* detection after immunosuppression cycle in a murine model of acute infection

Administration method	Number of mice with parasite rebound FBE (%)	Number of mice with quantifiable ***T. cruzi*** DNA (%)			Mice with parasitological cure (%)
		Blood	Skeletal muscle	Heart	
**GAV**	0 / 5 (0%)	0 / 5 (0%)	0 / 5 (0%) (4^a^)	1 / 5 (20%) (1^a^)	5 / 5 (100%)
**TIP**	0 / 5 (0%)	0 / 5 (0%)	0 / 5 (0%) (3^a^)	0 / 5 (0%)	5 / 5 (100%)

*FBE* fresh blood examination, *GAV* oral administration method by gavage, *TIP* oral administration method by pipette and tip

^a^ Number of samples with *T. cruzi* DNA detectable but below the quantization limit (Ct > 30)

## Discussion

This work was conducted to validate the efficacy and safety of a new, simple and low-cost method for daily oral drug administration using a murine model of *T. cruzi* acute infection. This method is potentially transferable to other rodent preclinical studies.

This refined strategy involves using a disposable tip and automatic pipette, which allows selecting final volume to the minimum required volume to dissolve the compound (e.g.: 20 or 50 μL), avoiding animal exposure to unnecessarily large volumes. Although this refined method is already in use in our protocols, we thought that it would be important to validate it in order to use it as a routine procedure for long-term oral drug administration in drug research and development.

To the best of our knowledge, up to now there were no studies evaluating compound efficacy in animal models of infectious or parasitological diseases while using a more refined oral administration method. Previous work reported morbi-mortality caused by repeated oral gavage drug administration in a murine model of *T. cruzi* infection, a situation that can potentially distort valuable results when studying new chemotherapies for Chagas disease and other diseases [11].

The refined oral administration method offers many advantages over conventional gavage administration, such as less animal handling time, less intensive operator training and, most important, reduced risk of upper respiratory and digestive tract damage or aspiration pneumonia in those protocols that require chronic oral compound administration. In addition, this method allows for accurate individual dose and volume tailoring.

A similar refined technique was described for 15 and 40 days-old rats previously trained for water deprivation with good acceptance [7]. Unlike this report, none of the animals in our study refused to drink BZ compound and there was no needed to train the animals nor submit to water deprivation period prior to dosing.

A potential disadvantage of this method is that treatment acceptance by the animals would be dependent on drug palatability, which may add variability to the efficacy depending on the taste of the drug and physicochemical characteristics of the administered solution with transient discomfort and a higher risk of substance loss. Although this situation was no observed in our work, palatability is a factor to consider prior to establishing a more refined technique procedure [12].

This acute model of *T. cruzi* infection was previously standardized and the employed VD strain shows high parasitaemia levels and high mortality rates in non-treated (NT) infected BALB/c mice [13]. Then, for this work, there was no NT group added since the aim was to present an alternative oral administration method with a reference drug. BZ was chosen for our study based on the proven efficacy and current use in experimental models and clinical cases of Chagas disease [10, 14].

BZ effectiveness (i.e. parasitological cure) was established both by the absence of rebound parasitaemia after immunosuppression and quantifiable parasite DNA detection in blood and target tissues assessed by qPCR. The absence of parasitaemia rebound was confirmed by qPCR and all blood samples were negative irrespective of the administration method.

Although *T. cruzi* DNA was detected in some samples from cardiac and skeletal muscle from both groups, the obtained signal was below the quantitation limit. It is important to note that VD strain shows a high tropism for skeletal muscle and therefore many *T. cruzi* DNA may be present at the site of infection [13]. Persistence of parasite DNA detectable but not quantifiable was consistent with parasitological cure in our model, as these results can be explained by the persistence of *T. cruzi* DNA in local phagocytes (as documented in previous studies [15] that may represent intra and/or extracellular granular antigens, abundant in heart and skeletal muscle during the acute phase of the infection [16].

CYP is a commonly used agent to establish immunodepletion in mice [10]. Two CYP injections at 150 mg/kg by intraperitoneal route are enough to produce severe leukopaenia [17]. Thereby, the CYP protocol used in our work can be considered enough to lead to parasitaemia rebound if there were any persisting parasites in sanctuary sites, such as heart and skeletal muscle due to BZ failure.

Previous studies in other experimental models have yielded similar observations. In a murine *Echinococcus multilocularis* experimental model, albendazole serum concentrations were similar when administered either by gavage or by voluntary ingestion in honey mixture, and a comparable antiparasitic activity achieved [2].

Although no pharmacokinetic analyses have been done yet, our results suggest than BZ efficacy is not affected by either administration method, and given the rapid absorption of oral BZ into circulation it is unlikely that significant pharmacokinetics differences would exist among both methods [18].

Due to the lack of similar studies, there was no previous data available on which to base an a priori power analysis. Moreover, our experimental outcome was a categorical variable (cured/no cured dichotomy), hampering sample size estimation beforehand. Accordingly, we assumed that the refined administration method should be as effective as the standard. Considering a complete treatment response in mice treated with BZ by gavage method (gold standard), it would be unacceptable to obtain biased results due to the administration method apart from the drug activity.

## Conclusions

As no significant differences were detected in drug efficacy between both administration methods, this refined technique consisting on administration with a tip and automatic pipette seems like a reliable method for BZ daily oral administration in an acute experimental murine model of *T. cruzi* infection while not affecting the experimental outcome and it could even be applied to similar murine models of related infectious or parasitic diseases.

## Material and methods

### Animals

Twenty-one days-old BALB/cJ male mice obtained from Animal Facilities (Faculty of Veterinary Sciences, University of Buenos Aires) were housed under conventional closed barriers at Dr. Ricardo Gutiérrez Buenos Aires Children’s Hospital animal facilities. Animals were acclimatized to new housing conditions and habituated to routine handling by trained personnel for 7 days prior to the experiment.

Mice were individually identified and housed in properly labelled, 600 cm^2^ polycarbonate cages at 5 mice per cage. Cages were filled with irradiated chip-bedding and changed once a week. Mice had ad libitum access to food (Rata-Ratón Cooperación®, Argentina) and water filtered by reverse osmosis. Macroenvironmental conditions included 12:12-h light: dark cycle (starting at 6 a.m.), a controlled temperature in a range of 20 to 22 °C, and humidity from 45 to 55%.

The study protocol was approved by the Institutional Animal Care and Use Committee from the Faculty of Veterinary Sciences – University of Buenos Aires (# Protocol: 2014/4).

### Experimental infection

Mice (28–35 days old; 20.12 ± 2.35 g) were infected with 1000 bloodstream trypomastigotes of *T. cruzi* VD strain, previously characterized [13] and maintained by serial passages in outbred CF-1 male mice. The inoculum was suspended in sterile RPMI-1640 medium and administered in a final volume of 0.2 mL per animal by intraperitoneal (ip) route. Treatment started at 12 days post infection (dpi), when all infected animals exhibited bloodstream trypomastigotes and it was randomly assigned as follows: GAV: BZ administered by gavage (*n* = 5); or TIP: BZ administered by a disposable tip and automatic pipette (*n* = 5).

### Drug and treatment administration

BZ commercial tablets (Radanil®, Roche) were crushed and suspended in a 0.25% solution of sodium carboxymethyl cellulose (Sigma-Aldrich Co.). Treatment was administered orally at a dose of 100 mg/kg/day for 20 consecutive days, between 10 and 11 a.m. All animals were weighed at least once a week, and drug doses adjusted for weights. Dose, length of treatment and route of administration were chosen based on published data [10].

**Fig. 3:**
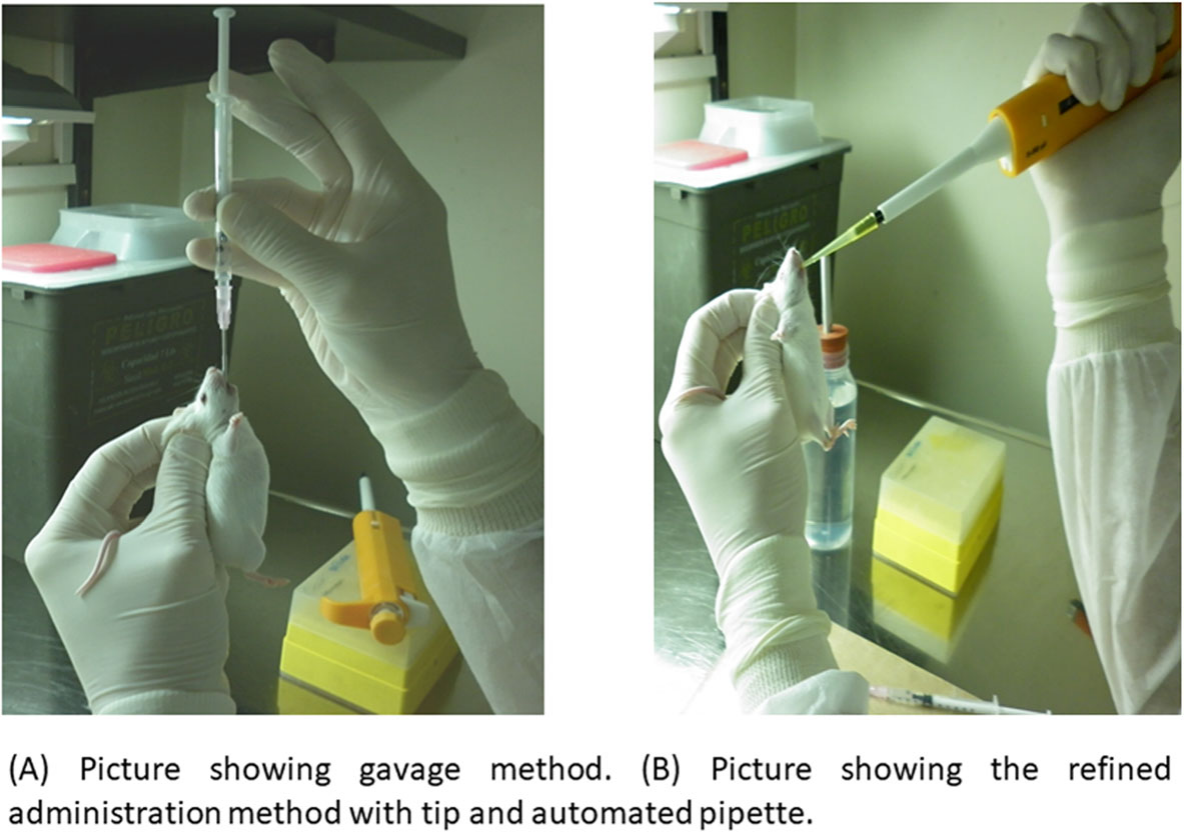
Mice receiving oral dosing of benznidazole

For gavage dosing, a disposable and flexible gavage with an 8–10 cm long 35 K nasogastric tube was prepared. This device was then coupled to a 1 mL syringe. The oral gavage technique was carried out as previously described [1] (Fig. 3a).

Pipette tip administration was performed with disposable tips (Kima®, Italy, #18170) and a 200 μL volume automatic pipette. This refined administration technique consisted in filling the pipette with the drug solution at the corresponding volume, along with physical restraining of the mouse with the least skilful hand and the pipette in the other hand. Then, the tip was slightly approached to the animal’s mouth and the volume was discharged slowly into the oral cavity, while gently pressing the hard palate with the tip to stimulate the swallowing reflex (Fig. 3b). The procedure was usually fast, and restraint was limited to less than 30 s since the volume to be dispensed was generally low (50–100 μL).

### Assessment of treatment response

Parasitaemia was evaluated since 7 dpi every day until it became negative for at least three consecutive days, using a modified Pizzi-Brener method [19–21]. A total of 5 μL of blood was taken by amputation of the tail tip. In the successive sampling, the clot was removed and the blood was extracted by milking the tail gently [22].

Then, the sample was compressed between a glass slide and an 18 × 18 mm coverslip and examined microscopically at × 400 magnification. The number of parasites per mL of blood was determined by counting trypomastigotes in 50 fields and then multiplying that number by a conversion factor (10^4^) to express the result in terms of trypomastigotes/mL. This technique allows detecting a minimum of 10,000 trypomastigotes/mL. Patent period (defined as the time elapsed between parasitaemia onset and parasitaemia clearance) and the number of doses to obtain parasite clearance were also recorded.

After treatment was completed, animals with undetectable parasitaemia were left without treatment for 10 days, with regular blood testing for re-emergence of bloodstream parasites, and then subjected to a cyclophosphamide (CYP)-based immunosuppression protocol to rule out parasite recurrence from sanctuary sites in tissues. Immunosuppression protocol consisted of 4 cyclophosphamide administrations (CYP) (Filaxis Laboratory, Buenos Aires, Argentina), at 200 mg/kg by ip route, separated by 7 days [23].

Parasitaemia was evaluated weekly during immunosuppression; finally, animals that remained negative were euthanized with sodium thiopental (Hipnopento®, Dr. Gray Products, Argentina) at 300 mg/kg by ip route. At the moment of euthanasia blood, skeletal and heart muscle were collected for *T. cruzi*-DNA testing by real-time PCR (qPCR).

### Preparation of peripheral blood and tissues for qPCR

One hundred microlitres of blood were obtained by submandibular vein puncture [24], collected in a sterile tube and mixed immediately with 200 μL of guanidine 6 M EDTA 0.2 M buffer. Rear legs skeletal and heart muscle samples (25–50 mg) were obtained with sterile scissors and were rinsed with sterile distilled water before collection into separated DNase-free tubes. Samples were stored at − 20 °C until processing.

### DNA preparation and q-PCR

Total genomic DNA was extracted from blood and tissues using the High Pure PCR Template Preparation Kit (Roche®). *T. cruzi* DNA amplification was performed on a StepOne PCR system (Applied Biosystems®) as described elsewhere [25]. Amplification product detection was performed using TaqMan® probe *cruzi 3* labelled with 5’FAM (6 – carboxyfluorescein) and 3’MGB (minor groove binder).

Each 48-well reaction plate contained two positive and two negative controls. Negative controls consisted of a reaction with *T. cruzi*-specific primers without DNA and with DNA extracted from blood or tissues from non-infected mice. PCR standard curves were generated by inoculating specimens with 8 × 10^5^ trypomastigotes of *T. cruzi* VD strain, followed by DNA extraction and serial dilutions. The standard curve allowed DNA quantification in the range of 1.6 × 10^0^ to 8 × 10^5^ parasite equivalents/mL. Amplification efficiencies were determined by StepOne Software v2.1 (Applied Biosystems®) by calculating efficiency (E) = 10^(− 1/slope)^. Parasite loads in individual specimens were calculated based on the standard curve included in each batch run.

### Experimental design and statistical analysis

No previous information was available to estimates the sample size and power analysis for this pilot study. Thus, the sample size was based on published suggestions to assess the efficacy of new compounds in mice models of *T. cruzi* infection [26]. Male mice were chosen since they are known to be more susceptible to *T. cruzi* infection than females, which would maximize the chances to observe a difference in response between interventions [27].

Each experimental group was composed of 4- and 5-weeks old mice at the moment of infection, and each cage was occupied by animals from different experimental groups, unequivocally identified.

Nonparametric tests were used when graphical inspection of data Shapiro-Wilk test indicated that data did not approximate to normal distribution and could not be transformed to meet assumptions for parametric analyses.

Non-parametric Mann-Whitney test was performed to assess differences in maximum parasitaemia values, and number of doses needed to achieve parasitaemia clearance between both administration methods, while differences in patent parasitaemia was analyzed with Mantel-Cox rank test. The statistical significance level was determined a priori at a *P-value* <0.05. Values are expressed as median and range. All analysis and graphics were performed with GraphPad Prism (GraphPad Prism, Version 5.03 for Windows, Graph- Pad Software, San Diego, CA, USA; www.graphpad.com).

## Data Availability

The datasets during and/or analyzed during the current study available from the corresponding author on reasonable request.
